# Supplementary material for: Transcriptional regulators of the Golli/myelin basic protein locus integrate additive and stealth activities
Source: PLoS Genet. 2020 Aug 13;16(8):e1008752. doi: 10.1371/journal.pgen.1008752 (PMC7446974; doi:10.1371/journal.pgen.1008752)
Supplement: S1 Fig — Electron micrographs were obtained from cross sections of the ventral medial cervical spinal cord from P90 WT and M3M5 mice (A and B at 640x) and (C and D at 3000x). The axon population in this domain ranges from small to large calibers. In the M3M5KO sample, axons of all calibers are typically ensheathed with compact myelin markedly thinner than normal although rare small calibre axons lacking compact myelin (*) were encountered. (PDF) [file pgen.1008752.s001.pdf]

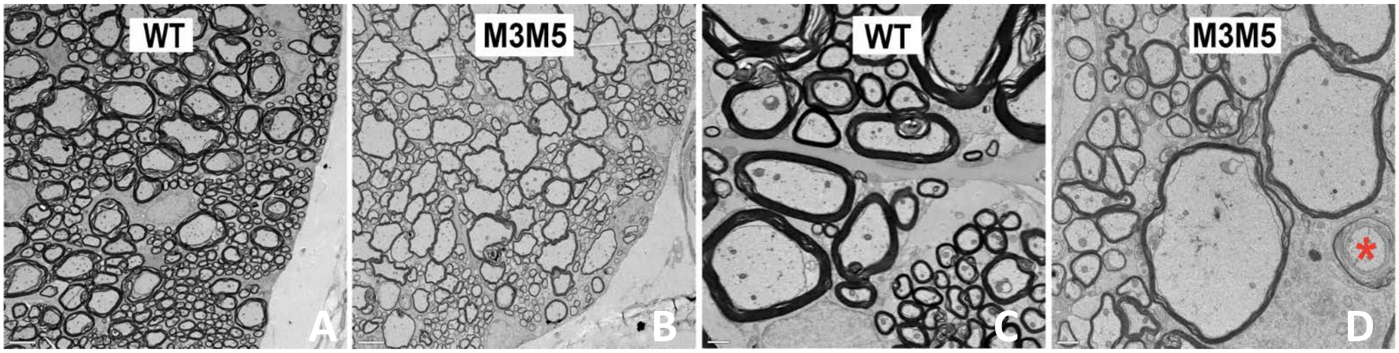

S1 Fig. Mice bearing the M3M5KO allele demonstrate CNS hypomyelination. Electron micrographs were obtained from cross sections of the ventral medial cervical spinal cord from P90 WT and M3M5 mice (**A** and **B** at 640x) and (**C** and **D** at 3000x). The axon population in this domain ranges from small to large calibers. In the M3M5KO sample, axons of all calibers are typically ensheathed with compact myelin markedly thinner than normal although rare small calibre axons lacking compact myelin (*) were encountered.
