# Supplementary material for: Transcriptional regulators of the Golli/myelin basic protein locus integrate additive and stealth activities
Source: PLoS Genet. 2020 Aug 13;16(8):e1008752. doi: 10.1371/journal.pgen.1008752 (PMC7446974; doi:10.1371/journal.pgen.1008752)
Supplement: S1 Table — The values are presented as % ± standard error of the mean. “*” and “**” represent p-values ≤ 0.05 and ≤ 0.01 respectively. n(F:M) represents the number of Female and Male mice from each genotype analyzed at each age. (PDF) [file pgen.1008752.s002.pdf]

% *Mbp/Gapdh* in spinal cord of mice										
	**P7**		**P14**		**P21**		**P30**		**P90**	
**Mouse line**	**% ± SEM**	**n(F:M)**	**% ± SEM**	**n(F:M)**	**% ± SEM**	**n(F:M)**	**% ± SEM**	**n(F:M)**	**% ± SEM**	**n(F:M)**
**WT**	100 ± 6%	2:4	100 ± 3%	3:3	100 ± 3%	3:3	100 ± 6%	3:3	100 ± 3%	3:3
**M3KO**	39 ± 2% **	3:3	59 ± 1% **	2:2	58 ± 3% **	3:3	63 ± 4% **	2:2	64 ± 3% **	2:4
**M3(225)KO**	-	-	85 ± 4% *	3:3	-	-	58 ± 3% **	3:3	82 ± 5% **	0:5
**M4KO**	-	-	92 ± 5%	2:2	-	-	103 ± 8%	2:2	81 ± 3% **	8:3
**M5KOΔ3.6kb**	53 ± 2% **	3:3	64 ± 2% **	3:3	58 ± 2% **	1:4	63 ± 2% **	3:3	79 ± 1% **	2:3
**M5KOΔ1kb**	-	-	62 ± 1% **	3:3	-	-	-	-	-	-
**M3M5KO**	15 ± 1% **	3:3	17 ± 2% **	2:2	23 ± 1% **	3:3	28 ± 1% **	3:3	38 ± 0% **	3:3
**M1EM3M5KO**	-	-	8 ± 0% **	3:2	14 ± 0% **	4:1	15 ± 1% **	4:2	25 ± 1% **	2:3

S1 Table. Relative *Mbp* mRNA analysis in spinal cord of enhancer knock-out mice at P7, P14, P21, P30 and P90. The values are presented as % ± standard error of the mean. “*” and “**” represent p-values ≤ 0.05 and ≤ 0.01 respectively. n(F:M) represents the number of Female and Male mice from each genotype analyzed at each age.
