# Supplementary material for: Transcriptional regulators of the Golli/myelin basic protein locus integrate additive and stealth activities
Source: PLoS Genet. 2020 Aug 13;16(8):e1008752. doi: 10.1371/journal.pgen.1008752 (PMC7446974; doi:10.1371/journal.pgen.1008752)
Supplement: S2 Table — The values are presented as % ± standard error of the mean. “*” and “**” represent p-values ≤ 0.05 and ≤ 0.01 respectively. n(F:M) represents the number of Female and Male mice from each genotype analyzed at each age. “#” indicates that sciatic nerves from two mice were combined for each sample. (PDF) [file pgen.1008752.s003.pdf]

% *Mbp/Gapdh* in sciatic nerve of mice												
	P4		P7		P14		P21		P30		P90	
**Mouse line**	**% ± SEM**	**n(F:M)**	**% ± SEM**	**n(F:M)**	**% ± SEM**	**n(F:M)**	**% ± SEM**	**n(F:M)**	**% ± SEM**	**n(F:M)**	**% ± SEM**	**n(F:M)**
**WT**	100 ± 5%	6#	100 ± 3%	3:3	100 ± 2%	4:5	100 ± 3%	3:3	100 ± 2%	8:1	100 ± 6%	1:2
**M3KO**	-	-	54 ± 3% **	3:2	81 ± 2% **	7:3	70 ± 4% **	3:3	75 ± 5% *	2:6	58 ± 5% **	2:4
**M3(225)KO**	-	-	-	-	99 ± 4%	3:3	-	-	95 ± 7%	2:3	-	-
**M4KO**	17 ± 1% **	6#	16 ± 1% **	3:3	17 ± 1% **	4:5	17 ± 1% **	3:3	23 ± 1% **	6:3	21 ± 2% **	2:1
**M5KOΔ3.6kb**	-	-	91 ± 5%	3:3	90 ± 3% *	5:5	81 ± 6% *	1:4	92 ± 7%	5:4	63 ± 5% **	4:2
**M3M5KO**	-	-	56 ± 1% **	3:3	73 ± 3% **	6:4	66 ± 5% **	3:3	96 ± 7%	5:5	44 ± 5% **	3:2
**M1EM3M5KO**	-	-	-	-	81 ± 4% **	3:2	83 ± 4% **	4:1	100 ± 10%	4:2	-	-

S2 Table. Relative *Mbp* mRNA analysis in sciatic nerve of enhancer knock-out mice at P4, P7, P14, P21, P30 and P90. The values are presented as % ± standard error of the mean. "*" and "**" represent p-values ≤ 0.05 and ≤ 0.01 respectively. n(F:M) represents the number of Female and Male mice from each genotype analyzed at each age. "#" indicates that sciatic nerves from two mice were combined for each sample.
