# Supplementary material for: Transcriptional regulators of the Golli/myelin basic protein locus integrate additive and stealth activities
Source: PLoS Genet. 2020 Aug 13;16(8):e1008752. doi: 10.1371/journal.pgen.1008752 (PMC7446974; doi:10.1371/journal.pgen.1008752)
Supplement: S3 Table — The values are presented as % ± standard error of the mean. “**” represent p-values ≤ 0.01. n(F:M) represents number of Female and Male mice analyzed. (PDF) [file pgen.1008752.s004.pdf]

% *Golli/Gapdh* in spinal cord of mice										
	P7		P14		P21		P30		P90	
**Mouse line**	**% ± SEM**	**n(F:M)**	**% ± SEM**	**n(F:M)**	**% ± SEM**	**n(F:M)**	**% ± SEM**	**n(F:M)**	**% ± SEM**	**n(F:M)**
**WT**	100 ± 3%	3:3	100 ± 3%	3:3	100 ± 4%	3:3	100 ± 11%	3:3	100 ± 3%	3:3
**M3KO**	7 ± 0% **	3:3	7 ± 0% **	2:2	8 ± 0% **	3:3	9 ± 1% **	2:2	11 ± 1% **	2:4
**M3(225)KO**	-	-	90 ± 8%	3:3	-	-	141 ± 5% **	3:3	66 ± 4% **	0:5
**M4KO**	-	-	97 ± 5%	2:2	-	-	114 ± 10%	2:2	94 ± 4%	8:3
**M5KOΔ3.6kb**	96 ± 3%	3:3	95 ± 2%	3:3	101 ± 4%	1:4	112 ± 6%	3:3	109 ± 6%	2:3
**M5KOΔ1kb**	-	-	90 ± 2%	3:3	-	-	-	-	-	-
**M3M5KO**	7 ± 0% **	3:3	7 ± 0% **	2:2	10 ± 0% **	3:3	8 ± 0% **	3:3	12 ± 0% **	3:3
**M1EM3M5KO**	-	-	7 ± 0% **	3:2	8 ± 1% **	4:1	9 ± 0% **	4:2	-	-

S3 Table. Relative *Golli* mRNA accumulation in spinal cord of enhancer knock-out mice at P7, P14, P21, P30 and P90. The values are presented as % ± standard error of the mean. “**” represent p-values ≤ 0.01. n(F:M) represents number of Female and Male mice analyzed.
