# Supplementary material for: Transcriptional regulators of the Golli/myelin basic protein locus integrate additive and stealth activities
Source: PLoS Genet. 2020 Aug 13;16(8):e1008752. doi: 10.1371/journal.pgen.1008752 (PMC7446974; doi:10.1371/journal.pgen.1008752)
Supplement: S4 Table — References are indicated by (). (PDF) [file pgen.1008752.s005.pdf]

Transcription factor/Histone modification	M1	M3	M4	M5	Other
SOX10 and H3K27ac CNS (52)	+	+	-	+	*Golli* intron 2 (chr18: 82683892-82684250)
MYRF CNS (51)	-	-	-	+	Upstream of *Golli* promoter (chr18: 82632652-82633185)
ZFP24 CNS (36) *	-	+	-	-	*Deleted in M3(225)KO allele
OLIG2 CNS (59)	-	+	+	+	Upstream of *Golli* promoter (chr18: 82631463-82633341)
					Golli intron 2 (chr18: 82683375-82685169)
					Golli intron 3 (chr18: 82709138-82709711)
BRG1 CNS (59)	-	-	+	+	*Golli* intron 2 (chr18: 82683435-82684673)
SOX10 and H3K27ac PNS (52)	+	+	+	+	*Mbp* exon 1, M1, M2 and further 5' (chr18: 82723004-82724191)
					Mbp intron 1 (chr18: 82726054-82727120)
					Mbp intron 2 (chr18: 82737024-82737165)
					Golli intron 3 (chr18: 82709138-82709514)
					Golli intron 3 (chr18: 82692199-82692338)
					Golli intron 3 (chr18: 82698659-82698983)
EGR2 PNS (34)	+	-	+	-	*Mbp* Intron 1 (chr18: 82726685-82726829)
					Mbp intron 1 (chr18: 82724199-82724328)
					Mbp Intron 2 (chr18: 82732298-82732374)
					Golli intron 3 (chr18: 82698797-82698900)
					Golli intron 3 (chr18: 82723514-82723563)

S4 Table. Oligodendrocyte and Schwann cell ChIP-Seq data relevant to the *Golli/Mbp* locus. References are indicated by ().
