# Supplementary material for: Transcriptional regulators of the Golli/myelin basic protein locus integrate additive and stealth activities
Source: PLoS Genet. 2020 Aug 13;16(8):e1008752. doi: 10.1371/journal.pgen.1008752 (PMC7446974; doi:10.1371/journal.pgen.1008752)
Supplement: S5 Table — (PDF) [file pgen.1008752.s006.pdf]

5’ target M4 + strand	CTCAGGCTGGCCAAGTATGT
5’ target M4 - strand	TCCCAGCCTACCCACATACT
3’ target M4 - strand	AACCACTTGACCTACTTGAG
3’ target M4 + strand	TGCCTCTCAAGTAGGTCAAG
5’ target M5 + strand	AGCATTCCACTAATTGTC
5’ target M5 + strand	ATACCAACAGTCAAGAAC
3’ target M5 - strand	CTTTGCAGGGTTCTCTAA
3’ target M5 + strand	TGTCTGCTCTATACTCTC
5’ large M5 - strand	CCGATCTCATGAGAAACGTC
5’ large M5 + strand	CACGTAATCTAAAGTATGTT
3’ large M5 + strand	GCATTCATTGTACATGGCTC
3’ large M5 + strand	CCTCATTCCCTCCTGGGTTT
5’ M1E + strand	GGCAAGCTAACGTGCTCCTT
3’ M1E - strand	CCTCTAGGCCTCGTACAGGC

S5 Table. sgRNA target sequences used to generate the KO mice.
