# Supplementary material for: Transcriptional regulators of the Golli/myelin basic protein locus integrate additive and stealth activities
Source: PLoS Genet. 2020 Aug 13;16(8):e1008752. doi: 10.1371/journal.pgen.1008752 (PMC7446974; doi:10.1371/journal.pgen.1008752)
Supplement: S6 Table — (PDF) [file pgen.1008752.s007.pdf]

Use	Oligo name	sequence
Generate all sgRNA templates by PCR	sgRNA	5'-AAAAG CACCG ACTCG GTGCC ACTTT TTCAA GTTGA TAACG GACTA GCCTT ATTTT AACTT GCTAT TTCTA GCTCT AAAAC-3'
With sgNAR, generate M4 L1 sgRNA template	M4_L_1	5'-ATTAA TACGA CTCAC TATAG GCTCA GGCTG GCCAAG TATGT GTTTT AGAGC TAGAA ATAGC AAG-3'
With sgNAR, generate M4 L3 sgRNA template	M4_L_3	5'-ATTAA TACGA CTCAC TATAG GTCCC AGCCT ACCCA CATAC TGTTT TAGAG CTAGA AATAG CAAG-3'
With sgNAR, generate M4 R5 sgRNA template	M4_R_5	5'-ATTAA TACGA CTCAC TATAG GCCTC TCAAG TAGGT CAAGG TTTTA GAGCT AGAAA TAGCA AG-3'
With sgNAR, generate M4 R7 sgRNA template	M4_R_7	5'-ATTAA TACGA CTCAC TATAG GAACC ACTTG ACCTA CTTGA GGTTT TAGAG CTAGA AATAG CAAG-3'
The following F and R pairs were annealed and cloned into DR274 to generate an sgRNA template		
M5_5_1F		5'-TAG GAG CAT TCC ACT AAT TGT-3'
M5_5_1R		5'-AAA CGA CAA TTA GTG GAA TGC T-3'
M5_5_2F		5'-TAG GAT ACC AAC AGT CAA GAA-3'
M5_5_2R		5'-AAA CGT TCT TGA CTG TTG GTA T-3'
M5_3_1F		5'-TAG GCT TTG CAG GGT TCT CTA A-3'
M5_3_1R		5'-AAA CTT AGA GAA CCC TGC AAA G-3'
M5_3_2F		5'-TAG GTG TCT GCT CTA TAC TCT C-3'
M5_3_2R		5'-AAA CGA GAG TAT AGA GCA GAC A-3'

M5_LONG_5_1F	5’-TAG GAT CTC ATG AGA AAC GTC-3’
M5_LONG_5_1R	5’-AAA CGA CGT TTC TCA TGA GAT-3’
M5_LONG-5_4F	5’-TAG GCG TAA TCT AAA GTA TGT T-3’
M5_LONG-5_4R	5’-AAA CAA CAT ACT TTA GAT TAC G-3’
M5_LONG_3_2F	5’-TAG GCA TTC ATT GTA CAT GGC TC-3’
M5_LONG_3_2R	5’-AAA CGA GCC ATG TAC AAT GAA TG-3’
M5_LONG_3_2F	5’-TAG GAA TAG CTC CGA AAC CC-3’
M5_LONG_3_2R	5’-AAA CGG GTT TCG GAG CTA TT-3’

crRNA_M1_left1	5’-/AltR1/rGrGrC rArArG rCrUrA rArCrG rUrGrC rUrCrC rUrUrG rUrUrU rUrArG rArGrC rUrArU rGrCrU /AltR2/-3’
crRNA_M1_left2	5’-/AltR1/rArUrA rArArA rCrGrC rArCrA rGrUrU rArCrG rCrGrG rUrUrU rUrArG rArGrC rUrArU rGrCrU /AltR2-3’
crRNA_M1_right1	5’-/AltR1/rGrCrC rUrGrU rArCrG rArGrG rCrCrU rArGrA rGrGrG rUrUrU rUrArG rArGrC rUrArU rGrCrU /AltR2/-3’
crRNA_M1_right2	5’-/AltR1/rArArG rCrGrG rCrUrU rUrGrU rCrCrC rCrCrU rCrUrG rUrUrU rUrArG rArGrC rUrArU rGrCrU/AltR2/-3’
225_crRNA	5’-/AltR1/rGrArG rUrGrG rGrCrG rGrArG rArUrG rUrUrU rGrCrG rUrUrU rUrArG rArGrC rUrArU rGrCrU /AltR2 -3’
225 ss HDR template (Ultramer DNA Oligo, IDT)	5’-GGA GCC ACT TTG GAG ACA CAG AGG CCT CTC TGT ATC TCA CAA ATA ACT GTA TTC AAA GGA CAC AAA GCC CAA CTG TTG TAA AAA TAT TAG TAT TCA GAT GCC GCC CAC TCA GTC TTG CCC TTT CCT TAG GCA CCC TGA ACT GAG TGG CCC -3’

S6 Table. Oligonucleotides used for CRISPR editing.
