# Supplementary material for: Transcriptional regulators of the Golli/myelin basic protein locus integrate additive and stealth activities
Source: PLoS Genet. 2020 Aug 13;16(8):e1008752. doi: 10.1371/journal.pgen.1008752 (PMC7446974; doi:10.1371/journal.pgen.1008752)
Supplement: Attachment — Submitted filename: Response to reviewers.pdf [file pgen.1008752.s008.pdf]

Dear editor,

We are very grateful for the thoughtful comments provided by all reviewers. We have addressed all issues raised and in particular, we have extensively revised the discussion to position our findings with those of previous investigations largely focused on the role of individual transcription factors. While 2 additional experiments were suggested, we now describe in-depth the extensive literature that addresses one such concern and we provide what we believe are compelling arguments that the present investigation achieves a milestone in its current scope. We believe it provides the necessary foundation from which the reviewer suggested experiment, along with many other fundamental questions, can now be designed and addressed. Beyond that, it provides unprecedented insight into the functional organization of *Golli/Mbp* regulatory sequences while revealing defining features of the super-enhancer domain in which our target enhancers are encompassed.

Beyond the above revisions, we have taken the opportunity during the review process to extend our observations to KO lines at older ages and this additional data is now included.

REVIEWER #1:

The manuscript by Bagheri and colleagues is on the characterization of regulatory elements within the *Golli/Mbp* locus of mice. Using CRISPR/Cas9-dependent genome editing the authors remove single sequence elements that have been previously characterized in transgenic mice as oligodendrocyte- or Schwann cell-specific enhancers and together form a super-enhancer. The authors find that the single enhancer elements exhibit the cell-

specific enhancer activity previously detected in standard transgenesis and can determine the relative contribution of each single enhancer to overall activity. In addition to this, there are a few bits of interesting additional information. For one, the authors find stealth activity for a Schwann cell-specific enhancer in oligodendrocytes and vice versa. Second, they find additive rather than synergistic activity of single enhancers in the super-enhancer structure. Finally, they observe a discrepancy in the activity of an enhancer truncation in standard transgenesis and CRISPR/Cas9-dependent genome editing.

The manuscript is well written and the data are clearly presented. The analysis on enhancer activity almost exclusively relies on RT-PCR of bulk RNA from spinal cord, sciatic nerve, thymus and retina, and is therefore unable to analyze whether changes of expression are homogeneous or heterogenous between cells of the same type. There is also the possibility that reduced expression in the normally expressing cell type is (partially) counteracted by ectopic expression generated as a consequence of genome editing. However, considering the wealth of information on this particular genomic locus, chances are very high that the authors‘ interpretations of RT-PCR on bulk RNA are correct.

We understand that our analysis, based on tissue homogenates, cannot take into account heterogeneity existing in oligodendrocyte sub-populations. This is an interesting consideration as such heterogeneity likely has wide implications for multiple aspects of oligodendrocyte biology itself potentially extending to critical circuit properties. We previously reported both temporal and spatial heterogeneity in the expression of reporter genes in Schwann cells and oligodendrocytes (1, 2, 48-50, 53, 58) and the literature is

rapidly accumulating further examples that now extend to the exciting domain of adult myelin plasticity. However, numerous observations support the view that the majority of the observations we report for each line are directly comparable between lines. First, we focused on the early myelinating spinal cord and took great care to recover samples from the identical cervical domain in all mice. Thus, even if this domain were to contain multiple oligodendrocyte sub-types, we expect them to be equally represented in all samples. Second, the density of oligodendrocytes in this specific domain has been carefully investigated during development of WT and fully amyelinated shiverer mice. Oligodendrocyte density was shown to be equivalent in mice of both genotypes from P10 through P30, the period in which the majority of our observations were made. This relationship supports the view that secondary consequences arising from the significantly milder hypomyelinating phenotypes encountered in the enhancer KO lines would have no effect on oligodendrocyte density. We now describe these observations in greater detail and assemble further arguments to support the following statement added to the text.

"Therefore, we interpret observations made on the different KO lines to reflect a valid estimate of the average *Mbp* mRNA accumulation realized by generally equivalent populations of oligodendrocytes and Schwann cells."

The reviewer's second point, that ectopic expression might be induced in the gene edits we introduced, a possibility of relevance to all gene edits, cannot be formally excluded by our present data. However, uniquely in-depth characterization of these same *Mbp* regulatory sequences, alone and in combination, in the context of reporter constructs has accumulated (1, 2, 48-50, 53, 58). As expected of randomly inserted transgenes, only line specific ectopic expression was observed and only in a typical proportion of expressing lines; a feature thought to be the result of enhancer trapping. In agreement with the reviewer's opinion

that the *Mbp* locus is well-characterized, no consistent ectopic expression program was assignable to any enhancer or enhancer combination. Thus, despite ample opportunity to encounter such activity, no evidence exists to suggest that that the mutant alleles derived for this investigation would drive expression in an ectopic manner.

To me the strength of the manuscript is that it summarizes a huge amount of mouse work and represents a heroic effort of settling once and for all the enhancer landscape of the *Golli/Mbp* locus. The obvious drawback of the manuscript is that it does not go beyond a standard expression analysis of the various enhancers. There is, for instance, no attempt to provide experimental evidence for the looping model provided in Fig. 6.

We understand that the present investigation does not provide any direct experimental evidence for the looping model we present. However, as that putative model accommodates much of our observations, we were motivated to include it as an aide in the design of future investigations. In the same light, we have added a supplementary table that presents the demonstrated engagement of other known TFs with the *Mbp* enhancers investigated here. It is our hope that the looping model along with the established enhancer deleted mouse lines will provide effective support for new investigations extending from TF engagement and chromatin conformation to super-enhancer formation and function. We now discuss more clearly that our extensive in vivo developmental functional analysis provides the essential foundation from which future analysis of such mechanisms can be effectively approached.

Taken everything together, I find the work highly informative and expect much added value to the field of glial biology. Therefore I am in favour of publication.

REVIEWER #2:

This manuscript reports an in vivo characterization of multiple enhancers for the mouse *Golli/Mbp* locus. The mouse *Golli/Mbp* locus derives two transcripts, *Golli* and *Mbp* mRNA, by two alternative promoters. Yet, the expression of *Golli* transcripts are ubiquitous, whereas the expression of *Mbp* transcripts is limited to nerve cells. According to previous studies, the transcription of this locus is regulated through the 5 enhancers (named M1 through M5) that are localized in the 20-kb upstream region of *Mbp* promoter, which are also the 3rd intron in the case of *Golli* transcripts. In this study, the authors have generated a series of deletion KO models targeting these enhancers, and subsequently characterized in vivo functions of these enhancers. They measured the expression levels of *Mbp* mRNA using the total RNA isolated from spinal cord and sciatic nerves, representing the oligodendrocytes and Schwann cells, respectively. They also performed a similar series of qRT-PCR analyses for *Golli* mRNA with the total RNA isolated form thymus and retina cells. According to the results, M3 and M5 enhancers are critical for the expression of *Mbp* in the oligodendrocytes, M4 enhancer for the expression of *Mbp* in the Schwann cells, and finally M3 enhancer for the expression of *Golli* in all the tissues tested. Besides these main observations, they also observed functional contributions by M3 and M5 enhancers to the expression of *Mbp* in the Schwann cells. This was unexpected, since M3 and M5 were previously shown to be non-functional in this nerve cell. Given these observations, the authors suggest that M3 and M5 enhancers may have a hidden or ‘stealth’ activity for the

transcription of *Mbp* in the Schwann cells. Also, the authors proposed a model involving various chromatin loop structures to explain how one enhancer (M3) could be involved in two different expression patterns: lineage-specific expression for *Mbp* transcripts and ubiquitous expression for *Golli* transcripts.

Overall, this study provides several new insights regarding the in vivo functions of several enhancers for the mouse *Golli/Mbp* locus. However, the authors need to consider the following criticisms to improve their manuscript for future publication. First, this manuscript seems to have a big, but unsubstantiated, jump from the conclusions from experimental results to the subsequent interpretations or claims. As described earlier, the immediate conclusions are simple and straightforward, which are confirming the predicted tissue-specific functions of three enhancers, M3, M4, and M5. Yet, the main claims include 1) the hidden or stealth activity of M3 and M5 in the unexpected cell type, the Schwann cells, and 2) potential interactions of the individual enhancers for the lineage-specific and ubiquitous expression of *Mbp* and *Golli* mRNA, respectively. As part of their claims, the authors propose a model involving chromatin loop formations, but without any actual data, such as 3C or other related results.

As in our response to reviewer #1, we fully agree that the present investigation does not include direct experimental evidence for the looping model. However, as our putative model accommodates many of our observations, we were motivated to include it as an aide to the design future investigations. It is our hope that the enhancer deleted mouse lines established for this investigation will provide effective support for new investigations on

TF engagement, chromatin conformation and super-enhancer formation. We expect the extensive in vivo and developmental functional analysis presented in this investigation will provide the essential foundation from which future analysis of such mechanisms can be most effectively pursued. Consequently, it was not our intention to present the tentative looping model as a claim or as a conclusion based upon our observations. In the revised version we have taken greater care to present the model in that light emphasizing our intention to present it as an effective aid in generating testable hypothesis.

They further suggest potential involvement of Sp1 and Yy1 transcription factors for these chromatin loop formations, but again without any actual demonstration of the binding of these transcription factors to this mouse locus. The authors need to first perform additional experiments and derive the relevant results to propose their model.

We did in fact refer to the relevant published data regarding the SP1 binding to this locus. We now have modified that paragraph for clarity.

"M1, M3, M4, M5 and the *Golli* proximal promoter all have predicted high affinity YY1 binding sites while only M3, M3(225) and the *Golli* proximal promoter have similarly high affinity SP1 binding sites (Jaspar; relative score (rs) > 90%) (100). However, lower affinity SP1 and YY1 motifs (80% < rs < 90%) exist in all. Accordingly, in oligodendrocytes, M3 and M5 interaction with the *Mbp* promoter could involve DNA-looping mediated by YY1 and/or SP1 dimerization (Fig 6A). While conditional KO of YY1 in oligodendrocytes leads to amyelination characteristic of MBP null shiverer mice, post-mitotic oligodendrocyte maturation unfortunately is blocked such that any direct role for YYI in *Mbp* transcription cannot be easily revealed in that model (88, 89). Notably, the M3(225)KO allele lacks the YYI motif and exhibits the same reduction seen in mice bearing the full M3KO mice at P30. However, higher levels of *Mbp* mRNA accumulation are observed at P14 and P90 and this partial restoration of enhancer-promoter interaction might be conferred through its retained SP1 motif (Fig 6A). These observations suggest that age-specific differences in capacity to promote inter-sequence interaction may exist. Indeed, the global methylation of DNA in both oligodendrocytes and Schwann cells is known to change during differentiation and myelination (101) and the DNA binding of YY1 is methylation sensitive (102). In addition, during myelin synthesis in oligodendrocytes SP1 becomes

phosphorylated via the PKC/Erk (103, 104); a pathway shown to increase its DNA binding capacity in smooth muscle (105). During this period, SP1 accumulation and binding to the *Mbp* promoter also increases (103, 104)."

As above, we use the model to draw attention to the distribution of Sp1 and Yy1 elements in the different enhancers and propose that their differential involvement could provide a possible strategy through which the enhancers might achieve their lineage and/or promoter specificities. We fully agree that testing this hypothesis will require direct experimental evidence and as above, we present the model believing it to be an effective tool from which the most meaningful experiments can be designed.

Second, the authors need to provide a more detailed description of the mouse *Golli/Mbp* locus, showing the exon structures of *Golli* and *Mbp* mRNA along with the positions of primer sets for qRT-PCR as part of Figure 1. According to the publically available data from UCSC genome browser, this locus is much larger than the one shown in Figure 1, and, furthermore, the upstream region and the other two introns of *Golli*, 1st and 2nd introns, seem to have many other potential enhancers that have never been mentioned in the manuscript. These are also likely involved in the transcriptional regulation of this locus, thus need to be discussed along with the 5 known enhancers.

Our investigation focused exclusively on known enhancers encompassed within the proximal 5' super-enhancer domain. In the event, results from the present analysis demonstrated that these confer the majority of the *Mbp* and *Golli* enhancing activity. However, we fully agree that for improved perspective, the full *Golli/Mbp* locus should be described in greater detail. In response to this suggestion we have improved Figure 1 by

including the domain extending from upstream of the *Golli* promoter through the *Mbp* gene. We amended the text to draw attention to the existence of further regions of interspecies conservation and even super-enhancer domains elsewhere in the *Golli/Mbp* locus. However, as the majority of the *Mbp* expression program is accounted for by the investigated M1 – M5 enhancers, activity realized through these additional putative regulatory domains must have minimal effect on *Mbp*. We believe the revised Figure 1 now presents the *Golli/Mbp* locus in a far more accessible manner.

Third, there are many run-on sentences throughout the text of this manuscript, which are very confusing. These long sentences need to be broken down properly for the better reading of the audience.

In the revised manuscript we attended to grammatical issues that introduced potential confusion and we believe the manuscript has now become considerably more accessible throughout.

REVIEWER #3:

The regulation of the *Mbp* gene in oligodendrocyte biology has been the focus of many studies in the last couple decades. While transcription factors regulating *Mbp* have been defined, there has been relatively little analysis of which enhancers are required at the endogenous locus for induction and maintenance of *Mbp* expression. The manuscript describes a series of mouse lines in which genome editing was used to delete enhancers of the *Mbp* gene. Some of the enhancers had been shown to regulate expression of this

myelin-associated gene in both oligodendrocytes and Schwann cells, but previous experiments had relied on transgenic reporter assays. The advance here is a combinatorial and comprehensive analysis of five *Mbp* enhancers using genome editing to create enhancer knockouts. The studies show the enhancer contributions to developmental regulation in both oligodendrocytes and Schwann cells along with regulation of the golli transcript. The analysis shows additive contributions of some specific enhancers in the oligodendrocyte lineage (including one activated by Myrf) and the predominance of one *Mbp* enhancer in Schwann cells. Relatively few previous papers have used genome editing to remove oligodendrocyte enhancers at endogenous loci, although one enhancer deletion (M3) had been previously described. The analysis is carefully done and represents a significant amount of effort. The developmental time course analysis provides important data during the active period of myelination and also during myelin maturity.

One technical concern is that some of the deletions cause hypomyelination due to fairly low *Mbp* levels, and there is some concern that changed expression levels may be due indirectly to changes in oligodendrocyte differentiation upon *Mbp* depletion. It would be strongly recommended to assess this by looking at other oligodendrocyte genes in the time course (e.g. Plp1, Cnp, Myrf) to assess this possibility. This issue should be considered in light of results of Aaker et al, ASN Neuro 2016, which should be cited and discussed.

As mRNA analysis was performed on whole tissue samples, a prerequisite of our data interpretation was that equally mature Schwann cells or oligodendrocytes were present in equivalent density in all lines. Unlike reporter gene based investigations in which all mice

bear normal *Mbp* alleles, the KO lines investigated here accumulated reduced levels of *Mbp* and/or *Golli* mRNAs. In the most affected line in which M1, M3, and M5 enhancers are deleted, *Mbp* mRNA accumulated to only 14% of WT at P21; a level similar to that observed with an earlier transgene preparation in which some CNS axons remained unmyelinated and the sheaths that were present were thin (REF). Should defective oligodendrocyte maturation and/or capacity to maintain myelin arise in the enhancer KO lines, it could secondarily disrupt the normal signalling pathways required to drive transcription of *Mbp*, and possibly other myelin protein encoding genes such as Plp. Such changes also might precipitate compensatory responses in the oligodendrocyte population. In that event, total mRNA accumulation in different lines would no longer represent comparable averages from cell populations of equivalent size or maturity.

The results of extensive earlier investigations directly address these issues and we have amended to discussion to include:

"Here, mRNA analysis was performed on whole tissue samples thus making the assay susceptible to multiple potential variables including changes in the density and/or differentiation state of relevant cell populations, compensatory feedback mechanisms and secondary changes beyond the transcriptional level. However, these potential caveats are largely dispelled by previous observations. Firstly, shiverer mice, despite their *Mbp* null status and fully amyelinated CNS, have a normal density of mature oligodendrocytes from P10 through P30, although a notable increase is observed at P60 (57). Consequently, in enhancer KO lines that demonstrate only reduced rather than totally absent *Mbp* mRNA accumulation, oligodendrocyte density is expected to be normal at least through P30; the period in which the majority of the present observations were made. However, whether an increase in oligodendrocyte density occurs by P90 remains to be determined. In a similar manner, the accumulation of other myelin gene products in the amyelinating oligodendrocytes of *Mbp* null shiverer mice, including the prominent *Plp* transcript and protein, show little change from WT (67, 68). Further, the rate of initiation of their truncated *Mbp* transcript also tracks closely that of WT (69). Thus, changes in cell density or major disruptions in myelin gene programming were not anticipated responses to the reduced *Mbp* mRNA accumulations imposed by the enhancer KOs. A similar relationship was expected to exist in the PNS; e.g., by P5 in WT mice, spinal root axons are heavily

myelinated and Schwann cell proliferation has ceased (10). Further, shiverer mice demonstrate only minor ultrastructural anomalies and mild thinning of PNS myelin sheaths (10, 13). Finally, the endogenous *Mbp* accumulation program observed in enhancer KO mice is reflected in the reporter accumulation programs observed in many transgenic lines that bear an intact *Golli/Mbp* allele (1, 2, 48-50). Therefore, we interpret observations made on the different KO lines to reflect a valid estimate of the average *Mbp* mRNA accumulation realized by generally equivalent populations of oligodendrocytes and Schwann cells."

Also raised by this reviewer, the Zfp24 KO mouse model is a particularly relevant example in which the expression of *Mbp* and CNS myelin formation is grossly disrupted. Should that phenotype be assignable directly to the interaction of Zfp24 with *Mbp* regulatory sequence (shown for M3 in Elbaz et al., 2018), it would provide important mechanistic insights into the control of *Mbp* transcription. In like manner, numerous other TF KO models have potential to make contributions to our understanding of the functional organization of the *Mbp* transcriptional regulatory mechanism (REFS). As such investigations provide critical insight into the regulatory network that underlies both specification and maturation of the oligodendrocyte lineage they are now referenced and discussed in the revised manuscript. However, we were initially dissuaded from including these important myelin-relevant observations in the original manuscript due to the involvement of these factors in early stages of lineage maturation or survival. Unlike *Mbp* that initiates expression near the final stage of oligodendrocyte maturation, coincident with the initiation of myelin elaboration, such factors often serve defining roles in lineage specification and/or earlier stages of lineage maturation. Thus, even in the case of Zfp24 where direct interaction with M3 is documented, it is not possible to assign the cause of the observed changes in *Mbp* transcription of Zfp24 KOs directly to changes in *Mbp* transcriptional regulation per se. Rather, as raised by this reviewer, consequences of disrupted lineage maturation preventing oligodendrocytes from reaching a mature *Mbp* expressing phenotype also could

play a role in the observed changes in *Mbp* expression. As this is an important point of critical relevance to oligodendrocyte and myelin developmental biology and we now discuss this directly in the revised discussion. We also include a summary of published ChIP-seq data for the regulatory sequences associated with this locus and present those findings in a readily accessible manner in a new table.

In addition, how does the decrease in *Mbp* expression observed by deleting the Myrf-bound M5 enhancer correlate with the decreased *Mbp* expression in the Myrf knockout? Since Myrf might have been thought to be required for *Mbp* induction, some explanation should be provided for the relatively modest effect of M5 deletion. Some *Mbp* regulators like Sox10/Olig2 are thought to be active throughout OPC/oligo development, while others like Myrf and Zfp24 are activated in myelinating oligos, so some mechanistic synthesis would be useful for the field.

We do draw attention to the capacity of *Mbp* to initiate transcription in the absence of the Myrf containing enhancer, M5, and refer to the modest reduction in *Mbp* expression observed in mice bearing the M5KO allele.

"However, KOs of oligodendrocyte or Schwann cell TFs often disrupt their maturation prior to expression of *Mbp* and myelin formation, and/or their survival and cell identity, thus making it difficult to assign a direct role for such TFs on *Mbp* transcription per se. An interesting example is MYRF, a presumptive master regulatory TF that, within the super-enhancer, only binds to M5 (51). In *Myrf* KO mice, maturation of oligodendrocyte was arrested affecting the expression of multiple myelin genes and preventing *Mbp* expression (27). However, the KO of M5 did not prevent *Mbp* expression but rather, resulted in only ~40% reduction in its accumulation."

The results are considered in the context of a looping model, which is appropriate although 3C data for *Mbp* in this cell type is not available. However, more recent super enhancer

studies have incorporated the condensate model of enhancer activity, which depends upon enhancer recruitment of a critical mass of factors that can induce partitioning of genes into transcriptionally active condensates. The Discussion can be revised to consider alternate models such as this.

We now discuss and reference the condensate model and also refer to it in our discussion of the future application of the enhancer KO models.

The specification of enhancers having "stealth" activity perhaps should be reconsidered as there does not have appear to be precise mechanistic definition of this property, other than enhancer activity was not detected in previous transgenic assays. Historically, some enhancers do not show activity unless multimerized so the absence of activity in a given transgenic context could depend on limitations of the original transgenic design.

This interesting issue could significantly limit the interpretation of our results and negate our conclusion that enhancers can exert stealth activity in a lineage where they show no autonomous activity. As we understand the specific concern, the autonomous activities assigned to enhancers in previous reporter based assays may not be accurate due to the extensive variables and limitations associated with the introduction of such constructs as randomly inserted transgenes. Such variables include the site of insertion, the number of construct copies and their configuration at the insertion site. The consequences of such variables are known to range from complete silencing through to massively enhanced expression levels to ectopic expression realized through enhancer trapping. Considering

that background, the reviewer suggests that the capacity of M3 bearing constructs to demonstrate autonomous low-level activity in Schwann cells could have gone undetected. In such a case, the M3 activity we detect in that lineage in the context of the endogenous locus would be a simple and accurate report of its autonomous activity.

While we fully agree that the reviewer has raised an important issue, we also believe that the exceptional depth of pre-existing insights on the autonomous activity of *Mbp* enhancers, including M3 and M4, convincingly support our interpretation. We recognize that we failed previously to describe the full extent of such pre-existing reporter construct data and without such access, a reader might not appreciate just how entirely unexpected any contribution of M3 to *Mbp* expression in Schwann cells and M4 to *Mbp* expression in oligodendrocytes was. We now provide what we hope is an adequate description of this essential background and explicitly reference our extensive publications that describe the uniquely robust data set from which the autonomous activity of M3 and M4 were defined. Specifically, we characterized the expression of relevant reporters in multiple transgenic lines, including those bearing multiple construct copies randomly inserted and others bearing a single construct copy inserted at a common and highly permissive site. These were all investigated for signal in the CNS and PNS at multiple ages using a highly sensitive ß-galactosidase histochemical and quantitative biochemical assays. Multiple examples of these preparations are included in those publications and of the constructs regulated by M3 through *Mbp* 5' flanking sequences, regardless of a random or controlled integration site, none expressed in Schwan cells. In contrast, but fully consistent with our stealth activity assignment, when M3 was ligated via a heterologous *hsp* minimal promoter to the

reporter, transient activity in Schwann cells was detected during a brief period of preweaning myelin elaboration. Further, such activity was assigned to M3 itself and not the minimal *hsp* promoter or the insertion site. Thus, in the context of an entirely artificial construct, and during a very brief period of Schwann cell maturation, M3 is capable of productively engaging relevant Schwann cell TFs. This capacity provides an intriguing hint of its quantitative contribution to expression of the endogenous *Mbp* locus as revealed in the present investigation. Similarly, M4, regardless of its configuration, never drove detectable expression in oligodendrocytes.

These combined observations provide a uniquely robust and consistent data set that we believe defines the full autonomous activities of M3 and M4. We now describe this body of work and its relevance to our conclusions in a clearer fashion.

REVIEWER #4

Bagheri et al. provide an in vivo functional dissection of the enhancers in the *Golli*/Myelin Basic protein locus in oligodendrocytes and Schwann cells from transgenic mice. Different enhancers in this locus were deleted by CRISPR-Cas9 in mouse and its effects of the expression of MBP and *Golli* in vivo in oligodendrocytes and Schwann cells was tested at different stages of mouse development. Different conclusions on the workings of these enhancers, that together form a super enhancer, were drawn.

The study provides interesting information on the regulation of an important locus. The conclusions are generally supported by the data and give important information that could not be drawn from earlier reporter studies of these enhancers. The results show mostly

additive effects of deletions of individual enhancers, which adds to the discussion as to whether super enhancers should be viewed as one functional unit or more as a collection of closely spaced but autonomously acting enhancers.

I support publication of this study in principle, but a few issues have to be addressed first.

a) The authors have to be more clear about their deletions. **M5KOF6 should be also in Fig. 2.** The M5KO line has a much bigger deletion in M5 than the M3M5 KO and M1EM3M5 KO line. The M5 deletion in the latter two lines is never tested in unison and its effect is therefore unknown. B) It would be good if the authors mark the different M5 deletions in their figures so the reader understands that they are different. C. It would also be good if the authors mention the number of mice used per experiment also in the materials and methods.

For added clarity we renamed two M5KO alleles throughout the revised manuscript; M5KO → M5KOΔ3.6kb and M5KOF6 → M5KOΔ1kb.

We chose not to include M5KOΔ1kb (formerly M5KOF6) in Fig. 2 presenting developmental expression because it was evaluated only at P14. At that age, as shown in Supplementary Table S1, both the M5KOΔ3.6kb (formerly M5KO) and M5KOΔ1kb alleles yield similar results.

We included the sample number range in all qRT-PCR experiments in the material and methods and refer to the supplemental tables for the exact number of each genotype for each tissue analyzed at every age.

The conclusion that the M5 multiple repeat regions do not influence function is not tested and should be removed. For this, one has to compare M5 deletions with and without (all) repeat regions and this was not done, for reasons unknown as it would make the experiments more clean.

As the reviewer notes, to test the role of M5 associated repeats a simple comparison between deletion of the conserved sequence with and without any flanking repeats would be required. While the 829 bp M5 deletion excludes all the repeat sequences we failed to derive that allele. However, a similar reduction in *Mbp* mRNA accumulation was observed with the M5KOΔ1kb and M5KOΔ3.6kb alleles at P14 (Supplementary Table S1). As the M5KOΔ3.6kb allele removes all repeats while the M5KOΔ1kb allele maintains only one, multiple repeat domains do not appear to influence the activity of M5 at this age. We have restated our interpretation in the result section in line with the reviewer's suggestion.

On the discussion, I agree that M3 has stealth activity in Schwann cells. However, the reduction MBP expression in M4 KO in oligodendrocytes is simply too few to conclude that M4 has stealth activity in oligodendrocytes. This conclusion should be removed.

Because the reduction in mRNA accumulation observed in oligodendrocytes bearing the M4KO allele was not large, and observed only in the P90 samples, the suggestion was to not include such activity in the stealth category. However, M4KO mice accumulated 81% of WT *Mbp* mRNA values at P90 which is close to the value observed for M5KO mice (79% of WT). As any reduction was unexpected, we confirmed this small difference by analysis of a larger

than normal sample number that provided compelling statistical significance. We therefore feel obliged to include it in the category of stealth activity, but we take care to note that the stealth activity assigned to M4 is observed only in mature mice.

M5 KO has a small effect on MBP expression in Schwann cells but as its reporter activity was not tested, if I understand correctly, the hint that it also has stealth activity should be removed.

Direct insight into the expression programs conferred by M5 regulated reporter constructs in transgenic mice is not yet available. Consequently, the autonomous activity of M5 might extend to Schwann cells. In such a case, the reduced accumulation of *Mbp* mRNA observed in that lineage in M5KO mice would be a simple reflection of its autonomous activity. Due to the many programming features of M5 with similarities to M3, we raised the possibility that it might exert its observed activity in Schwann cells via stealth activity. However, the reviewer is correct that our hint of such activity was too speculative. In response, we have taken care to explain why stealth activity cannot yet be assigned and state clearly that the autonomous or stealth origin of such M5 activity remains to be investigated.

Summary:

We are grateful for the obviously informed considerations provided by all reviewers and we believe our revisions address all comments and suggestions.

A recurring comment was that our assessment of enhancer function was based only upon qRT-PCR analysis of *Mbp* mRNA accumulation within tissue homogenates. Consequently, numerous scenarios involving different oligodendrocyte density or maturation in the different lines could complicate our interpretation of the quantitative results. We now reference the extensive published background that largely dispels such concerns. *Mbp* is possibly the last myelin gene to be expressed during the post-mitotic maturation of oligodendrocytes and its total loss has remarkably little effect on the expression of other myelin genes, unlike the dramatic consequences of deleting many critical TFs. Of direct relevance, *Mbp* transcript initiation, inferred from the signal generated by the intact 5' portion of the transcript, is normal through pre-weaning development clearly demonstrating that no *Mbp* specific feedback mechanism operates during primary myelin elaboration.

A critical issue was the possibility that we could have missed autonomous enhancer functions in both the oligodendrocyte and Schwann cell lineage in our previous investigations of reporter constructs. If so, there would be no reason to invoke stealth activity in the apparently non-targeted lineage. Our extensive published record suggests that the full autonomous activities of the relevant enhancers are well established. Such previous investigations include the functional characterization of scores of *Mbp* regulated reporter constructs inserted both randomly in variable copy number and in controlled single copy preparations. In the revised manuscript this critical background is more readily accessible.

Although we provide no direct evidence for the proposed looping model, we are encouraged by the recognition of several reviewers that in its present form, the investigation represents a unique and formidable attack on a previously elusive topic and considerably advances our understanding of myelin gene transcriptional regulation. We fully agree that the reviewer proposed investigations are logical next steps in investigating how lineage specificities are allotted. Other obvious lines of investigation have potential to greatly increase our understanding of super-enhancer formation and function. However, we believe that the tentative model, the enhancer-relevant TF binding data now included (Supplementary Table S4) and the available KO models themselves will support next steps that we expect the wider myelin and transcriptional regulation communities will consider and possibly exploit.

Finally, we believe our careful edit of the discussion addresses all of the remaining reviewer issues. In responding to their comments, we believe that the manuscript is markedly more accessible and considerably more integrated into the broad literature on the transcriptional regulation of myelin genes and oligodendrocyte and Schwann cell biology.

Thank you for your continued consideration,

Alan C. Peterson
